# Supplementary material for: The Spatial Organization of Bacterial Transcriptional Regulatory Networks
Source: Microorganisms. 2022 Nov 30;10(12):2366. doi: 10.3390/microorganisms10122366 (PMC9787925; doi:10.3390/microorganisms10122366)
Supplement: Supplementary file 1 [file microorganisms-10-02366-s001.zip › Supplementary_Figures.pdf]

Supplementary Figures

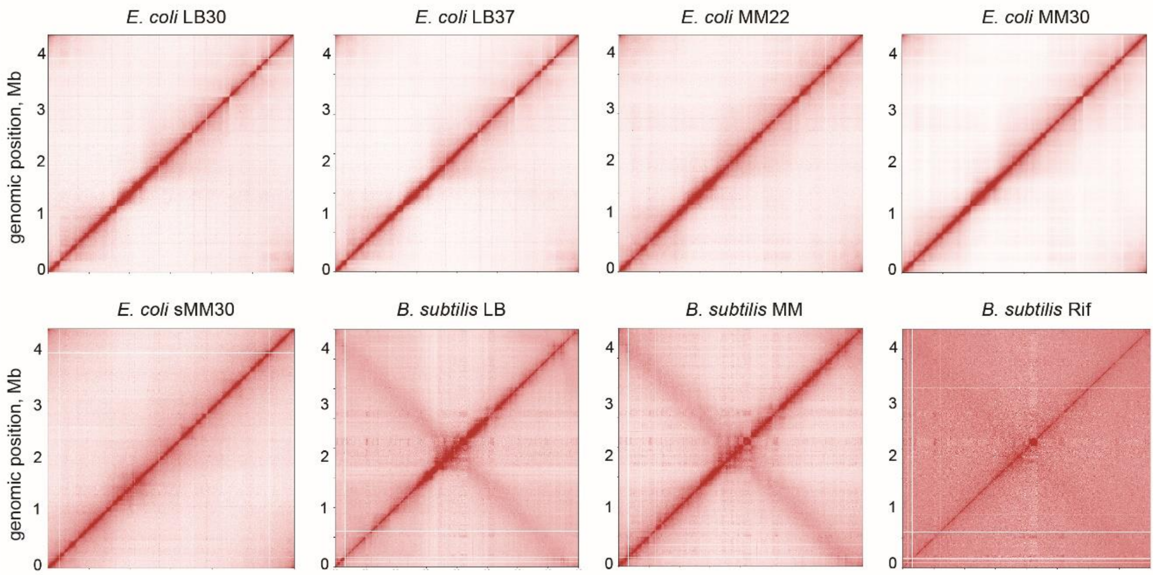

Figure S1. The normalized chromatin interaction frequency matrixes used in this study.

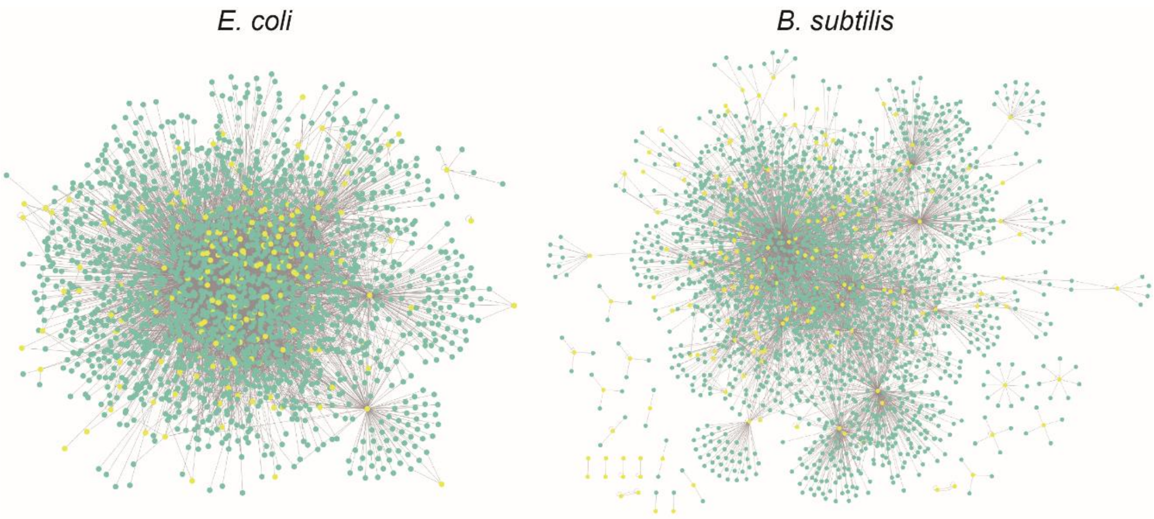

Figure S2. The reconstructed transcriptional regulatory networks (TRNs) of *Escherichia coli* and *Bacillus subtilis.* Nodes of yellow color are transcription factors.

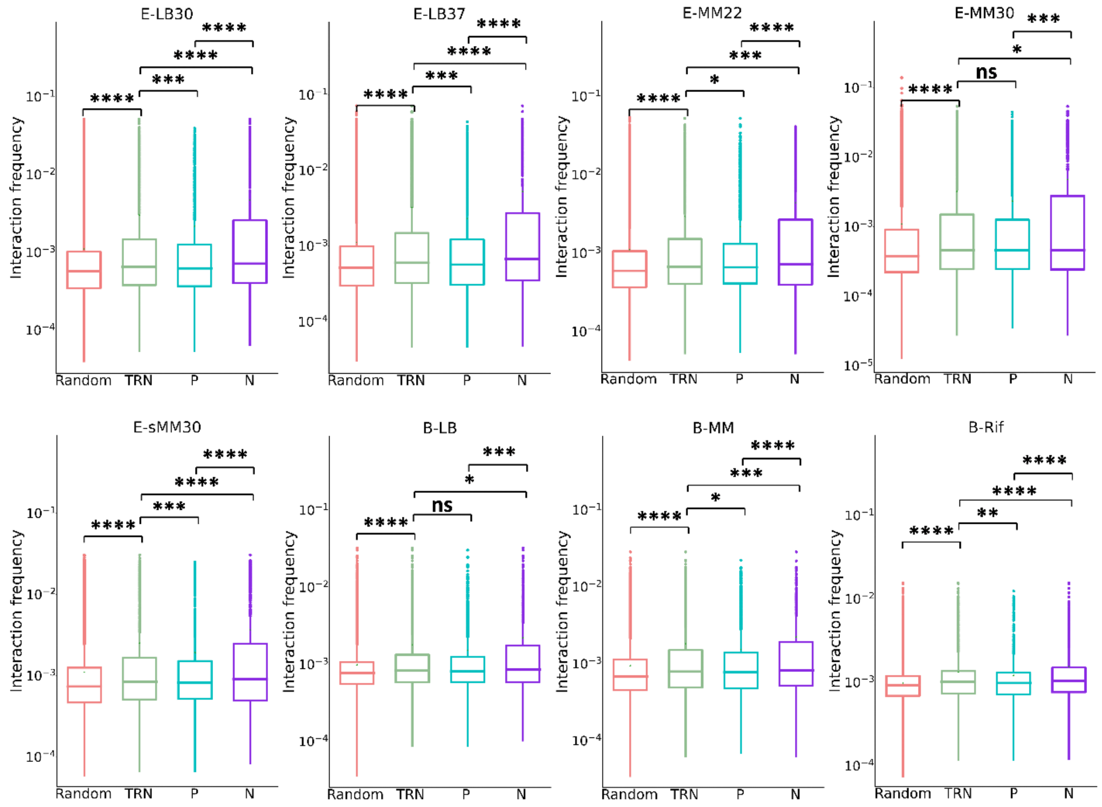

Figure S3. Comparison of chromatin interaction frequency in global TRN. Here "Random" means the random pairing of regulator-target genes in TRN. See also **Figure 1** in the main text for details. The symbols on the figure indicate statistical significance levels: ns: $p > 0.05$; *: $p \leq 0.05$; **: $p \leq 0.01$; ***: $p \leq 0.001$; ****: $p \leq 0.0001$.

Key Nodes	E-LB30	E-LB37	E-MM22	E-MM30	E-sMM30	B-LB	B-MM	B-Rif
In-Hub-I	> 1.74E-09	> 1.55E-05	> 6.88E-09	> 9.23E-07	> 5.95E-08	> 7.05E-06	> 0.0207	= 0.1293
In-Hub-O	= 0.9408	= 0.9676	< 1.93E-05	< 0.0003	< 0.0229	= 0.4985	= 0.2325	< 0.0057
Out-Hub-I	> 0.0450	> 0.0392	= 0.1414	= 0.1333	= 0.1968	> 0.0001	> 0.0005	> 0.0020
Out-Hub-O	< 1.73E-15	< 1.07E-16	< 2.27E-15	< 8.27E-15	< 1.02E-14	< 3.30E-17	< 2.90E-17	< 1.87E-14
Bottleneck-I	> 0.0016	> 0.0040	> 0.0132	> 0.0155	> 0.0095	> 0.0008	> 0.0003	> 4.38E-05
Bottleneck-O	< 2.25E-14	< 4.86E-15	< 2.83E-14	< 1.21E-13	< 4.46E-14	< 3.46E-12	< 1.97E-14	< 4.03E-12
Center-I	> 0.0040	= 0.3958	= 0.5635	= 0.1122	> 0.0039	NA	NA	NA
Center-O	> 0.0002	> 0.0420	= 0.4143	= 0.6267	> 0.0063	< 0.0067	= 0.2043	= 0.5624

Figure S4. Chromatin interaction frequencies of central nodes compared with TRN by using Wilcoxon rank sum tests. "-I" and "-O" after In-Hub, Out-Hub, Bottleneck, Center represent the chromatin interaction "intra" the 4 kinds of central nodes and the chromatin interaction between the 4 kinds of central nodes and "other" genes, respectively. ">", "<", "=" represent the chromatin interaction frequency is higher, lower, of no significant difference compared with that of TRN, respectively; the number below them are p-values in Wilcoxon rank sum tests for statistical significance. Red: significantly higher than TRN; Green: significantly lower than TRN.

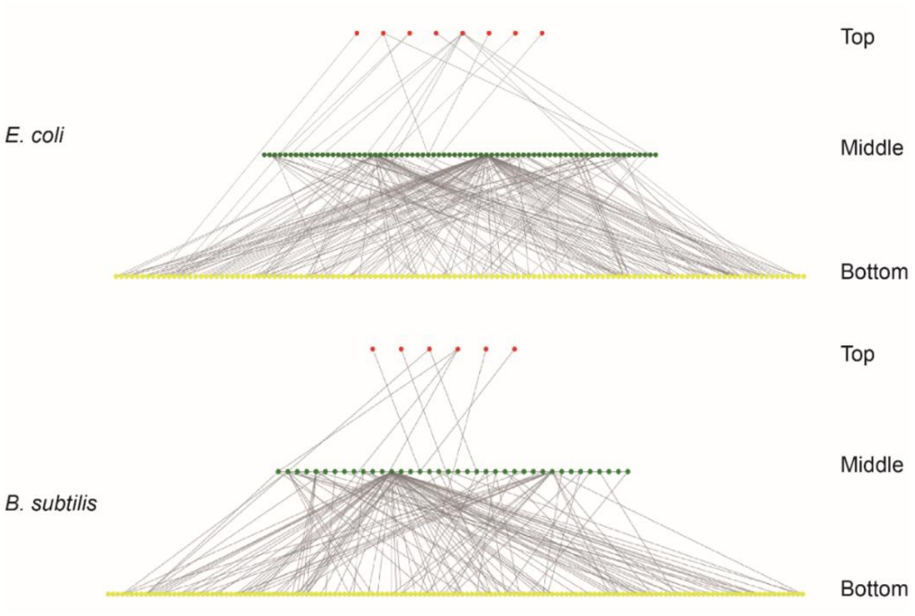

Figure S5. The hierarchical structures of *Escherichia coli* and *Bacillus subtilis* TRNs.

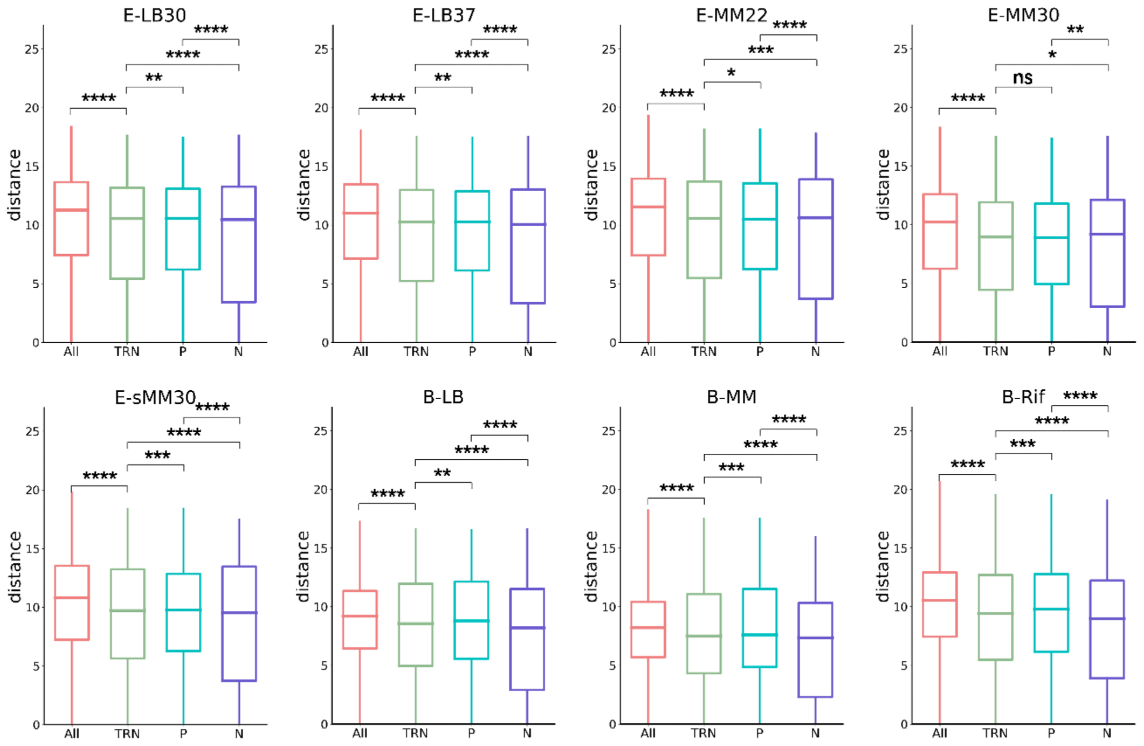

Figure S6. Comparison of spatial distances between gene pairs in global TRN. In almost all culture conditions, the spatial distances within TRN (denoted as TRN) are significantly shorter than those between all the gene pairs in the whole genome (denoted as All); the spatial distance of positive regulation (P) is significantly longer than TRN, while the spatial distance of negative regulation (N) is significantly shorter than TRN. The symbols on the figure indicate statistical significance levels: ns: $p > 0.05$; *: $p \leq 0.05$; **: $p \leq 0.01$; ***: $p \leq 0.001$; ****: $p \leq 0.0001$.

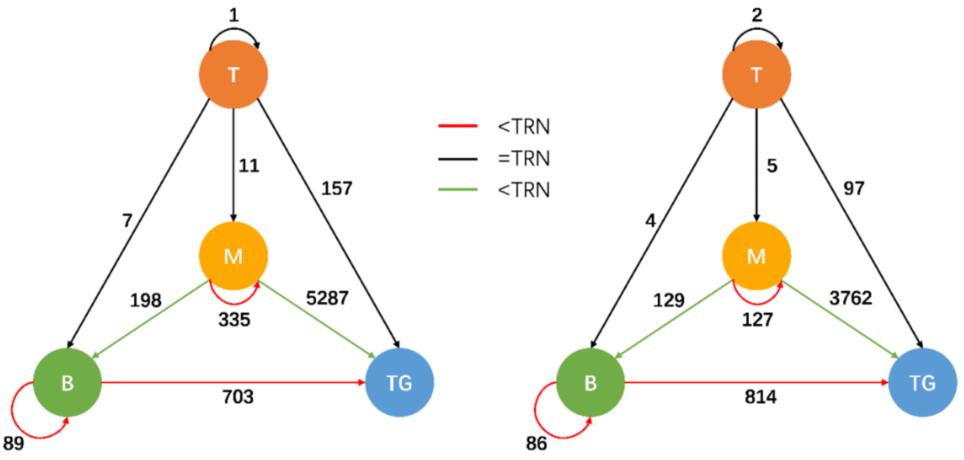

Figure S7. The spatial organization of TRN hierarchy based on 3D distance. The four nodes T, M, B and TG represent the Top, Middle, Bottom and Target layers in the hierarchy, respectively. The numbers on the edges represent the numbers of regulatory relationships within or between layers. The color of edge represents the result of comparing the spatial distance between the gene pairs of the edge with TRN. Significance level: $p < 0.05$.

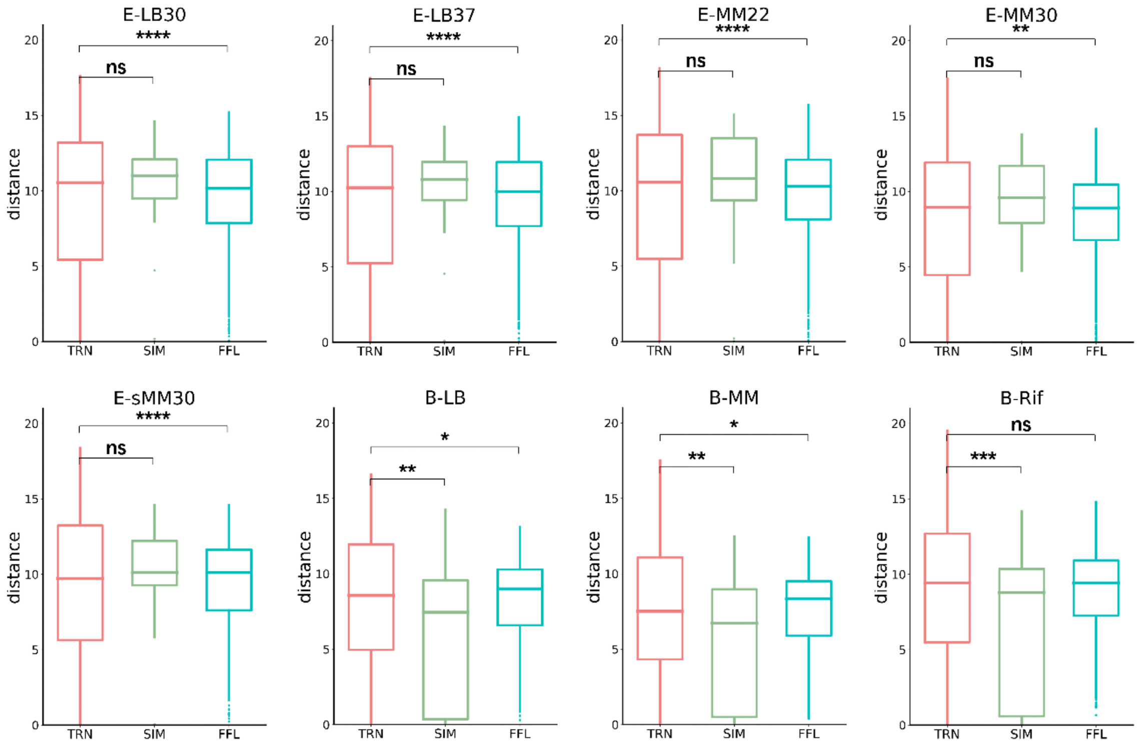

Figure S8. Comparison of spatial distances between gene pairs in network motifs with TRN. In the five culture conditions of *E. coli*, the spatial distance of FFL is significantly shorter than that of TRN and the spatial distance of SIM is of no significant difference from TRN. For most cases in *B. subtilis*, the spatial distances between gene pairs of both kinds of network motifs are significantly shorter than that of TRN. The symbols on the figure indicate statistical significance levels: ns: $p > 0.05$; *: $p \leq 0.05$; **: $p \leq 0.01$; ***: $p \leq 0.001$; ****: $p \leq 0.0001$.

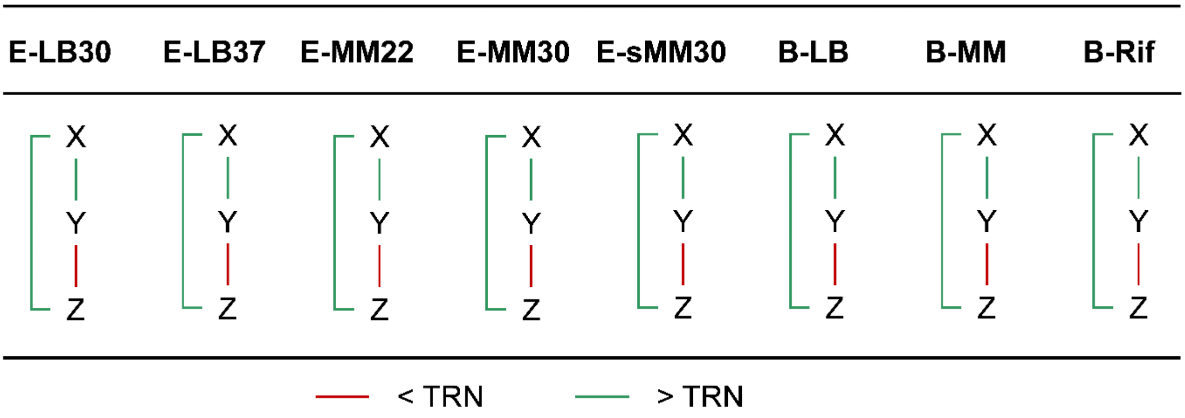

Figure S9. Comparison of spatial distances between gene pairs in FFL edges with TRN. In all cases, the spatial distance between X and Y/Z in feed-forward loop is significantly longer than TRN, while the spatial distance between Y and Z is significantly shorter than TRN. Significance level: $p < 0.05$.

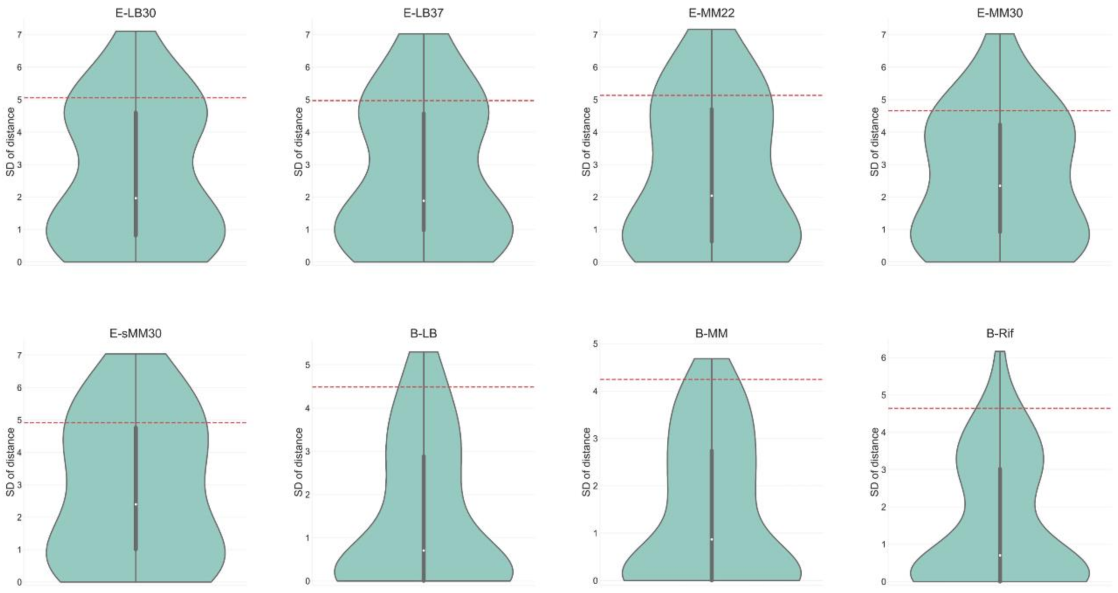

Figure S10. The distribution of standard deviation (SD) of spatial distance within SIMs (violin plot) and its comparison with that of TRN (red dashed line). The SD of spatial distance within SIMs is apparently lower than that of TRN, indicating lower dispersion and higher uniformity of spatial distance within SIMs.
